# Fear of Backlash Moderates Female Senior Executives' Communion (but Not Agency) as Compared to Female Lecturers

**DOI:** 10.3389/fpsyg.2021.520590

**Published:** 2021-10-29

**Authors:** Xiao Tan, Bin Zuo, Fangfang Wen, Zhijie Xie, Shijie Song

**Affiliations:** ^1^Institute of Educational Sciences, Hubei University of Education, Wuhan, China; ^2^Department of Psychology, Center for Studies of Social Psychology, Central China Normal University, Wuhan, China; ^3^School of Psychology, Xinxiang Medical University, Xinxiang, China

**Keywords:** female senior executive, communion, agency, moderation effect, fear of backlash

## Abstract

Today, many women work in occupational roles that had once been dominated by men (e.g., senior business executives). However, expectations on senior executives to be agentic (e.g., assertive, dominant) may conflict with prescriptive stereotypes about women to be communal (e.g., helpful, warm). According to this double-bind dilemma, female senior executives get criticized for lacking either agency or communion as both dimensions can be perceived as posing a tradeoff. We hypothesize that female senior executives report higher levels of agency and lower levels of communion than women in a more neutral role (e.g., lecturers) due to the perceived requirements of these occupational roles. In Study 1, *N* = 212 students rated adjectives on their desirability for men vs. women in Chinese society. They rated agentic characteristics as more desirable for men and communal characteristics as more desirable for women. Studies 2 and 3 used this material. Study 2 randomly assigned *N* = 207 female students to the role of a senior executive vs. lecturer. Study 3 was conducted with *N* = 202 female role occupants (96 senior executives, 106 lecturers). As expected, female senior executives reported higher levels of agency and lower levels of communion than female lecturers in both studies. Some women may be particularly aware of the above-mentioned double-bind dilemma and may be more worried about the potential backlash than others. They may attempt to reconcile occupational demands (i.e., higher agency, lower communion) with prescriptive gender stereotypes (i.e., lower agency, higher communion). We, therefore, explored whether fear of backlash attenuates the effect of the type of role of women (senior executives vs. lecturers) on agency and communion. Indeed, we found that senior executives who were particularly worried about backlash reported almost as much communion as lecturers did. In contrast, senior executives consistently reported higher levels of agency than lecturers regardless of their fear of backlash. The present study documents prescriptive gender stereotypes in China, how women differ as a function of their occupational roles, and how fear of backlash may motivate female senior executives to reconcile having high levels of both agency and communion.

## Introduction

### Agency-Communion Tensions and the Double-Bind Dilemma of Women in the Workplace

“*Women who attempt to fit themselves into a managerial role by acting like men are forced to behave in a sexually dissonant way. They risk being characterized as ‘too aggressive.’ Yet women who act like ladies, speaking indirectly and with concern for others, risk being seen as ‘ineffective.’”*

*Nancy Nichole*

Although traditional gender roles still exist in China, there are more and more women who break the glass ceiling and occupy the senior executive role. According to a 2016 international business report, 30% of senior business executives in China were women (Brosnan, 2016). In-depth interviews research has revealed that the double-bind dilemma is one of the barriers to the advancement for Chinese female managers and also female managers from other Asian countries (Peus et al., 2015). The double-bind dilemma is a dilemma that women in counter-stereotypical roles, especially in a managerial role, hardly meet competing demands, and they are usually perceived as either agentic or communal, but rarely both (Eagly and Carli, 2007).

The double-bind dilemma is rooted in the prejudice based on the prescriptive gender stereotype, produce agency-communion tensions (Heilman, 2012; Rudman and Glick, 2012). The prescriptive gender stereotypes capture beliefs about socially desirable expectations of the behavior of men and women (Burgess and Borgida, 1999; Prentice and Carranza, 2002; Ellemers, 2018). In the social stereotypical ideal, men are expected to possess more self-centered agentic attributes (e.g., being strong, dominant, and assertive), whereas women are expected to possess more other-oriented communal attributes (e.g., being kind, caring, nurturing, and communicative) (Bakan, 1966; Bem, 1974; Koenig, 2018; Hentschel et al., 2019). Meanwhile, people tend to have fixed beliefs about occupations men and women usually do and associate stereotypes of men and women with particular features of a job description (Eagly and Steffen, 1984). For example, people usually associate good managers with agentic attributes because they think men make successful managers (Schein et al., 1996; Koenig et al., 2011).

Therefore, the agency and communion tension emerged in the double-bind dilemma because agency and communion naturally seem like opposite traits and women will receive an adverse consequence for being either agentic or communal. For instance, female leaders show communal, like caring and warmth, typically elicits a “lack of fit” perception (Heilman, 2012; Zheng et al., 2018a). In this “lack of fit” perception, communal attributes are viewed as inconsistent with those traditionally associated with success in male-dominant occupations and positions (Heilman, 1983, 2001, 2012). Contrarily, women displaying agentic behaviors in the double-bind dilemma will potentially elicit social rejection, hiring discrimination, and sabotage. Data from several studies suggest that women displaying agentic behavior, including self-promotion and dominant behavior, suffer a backlash, such as being perceived as less likable, less hireable, or less promotable, as well as other social and economic penalties (Rudman, 1998; Moss-Racusin and Rudman, 2010; Rudman et al., 2012a; Williams and Tiedens, 2016; Schaumberg and Flynn, 2017; Hentschel et al., 2018).

### Women Coping With Agency-Communion Tensions in the Double-Bind Dilemma

The double-bind dilemma produced barriers for the counter-stereotypical career path of women, and women have to cope with potential agency-communion tensions. Multiple researchers have predicted the coping strategies of women in inconsistent ways. Some researchers have argued that counter-stereotypical women would strengthen agentic behavior and avoid communal behavior to decrease gender conformity, whereas others have suggested that they would avoid agentic behavior and exhibit more feminine and communal behavior to increase gender conformity.

On the one hand, many researchers found that women in the double-bind dilemma may try to be consistent with the occupation stereotype and avoid gender conformity by enhancing agency and decrease communion at work. The qualitative research by Ely (1995) found that compared with women in gender-balanced law firms, women working in male-dominant law firms tend to devalue femininity by exaggerating psychological and behavioral gender differences, associating masculinity with career success, and exclusive femininity traits from the attributes that firms valued. She identified that half of the women participants in her study from male-dominant law firms as “accommodators,” who adopt masculine characteristics and avoid feminine ones to display the attributes demanded by the firm. Further, Roberts (2005) suggested that avoiding gender conformity in the counters-stereotypical role is one of the impression-management strategies helping people increase social mobility by changing the social categories assigned to people. In this strategy, people will emphasize the similarity between themselves and the target social identity (e.g., senior executives) and reduce their similarity to their original social identity (e.g., women) to assimilate into the new target social identity (Dovidio et al., 2000; Ellemers et al., 2002).

On the contrary, other researchers illustrated that women in the double-bind dilemma would increase gender conformity by diminishing agency and reinforcing communion at work. Previous research has reported that women are more likely to engage in protective strategies by doing more gender-congruent behavior to avoid negative impressions, such as hedging and apologizing (Carli, 1990; Lee et al., 1999). Carli demonstrated that women in the discussion used more disclaimers, hedges, tag questions, and spoke more tentatively than men in general, and women had more influence on men when they spoke tentatively in a discussion or speech (Carli, 1990). In an experiment by Rudman and Fairchild (2004), it has been suggested that gender deviants would behave more in conformity with their gender, and counter-stereotypical women would display more femininity to defend themselves. Roberts (2005) also stated that in confirmation strategy of impression management, women could gain higher positive influence by confirming gender-congruent behavior, such as women may receive positive feedback by acting as mothers by showing communal attributes in organizations in male-dominant occupations (Sheppard, 1989; Pierce, 1995; Kaiser and Miller, 2001). In some counter-stereotypical contexts, when women perceive certain gender expectations, they may engage in gender conformity by a decrease in agentic behavior even if it harms the career success of women. For instance, researchers found that women would interfere with their self-promotion behavior in job promotion situations and rate their self-promotion less successful than men (Moss-Racusin and Rudman, 2010). This kind of phenomenon is also found in salary negotiations. Women use less competitive negotiation strategies and are worried that they might be viewed as too confident (Amanatullah and Morris, 2010).

### Fear of Backlash Moderates How Women Cope With Agency-Communion Tensions

Men and especially women who violate prescriptive gender stereotypes will encounter the “backlash effect” (Rudman et al., 2012b). The concept of backlash against women was first used in journalist Faludi's book to describe the negative reactions toward women in the progress of gaining equal rights with men (Faludi, 1991). Rudman defined the backlash effect as social and economic penalties for counter-stereotypical behavior of men and women, such as social rejection, hiring discrimination, and sabotage in the double-bind dilemma (Liu and Zuo, 2006; Rudman et al., 2012b). The fear of backlash happens when people in counter-stereotypical roles are aware that there will be backlash going against them (Rudman and Fairchild, 2004).

Several attempts have been made to explore how fear of backlash influences the counter-stereotypical behavior of an individual (especially for women in the double-bind dilemma). In Rudman's backlash and stereotype maintenance model, she proposed that fear of backlash would cause recovery strategies, including hiding atypical behavior, deception, and increased norm conformity under the condition of expectancy violation (Rudman et al., 2012b). Researchers also conducted some experiments to discover the specific role of fear of backlash. In virtual competition game experiments, people who are in the stereotype violation condition would hide their success in the game which is mediated by fear of backlash (Rudman and Fairchild, 2004). In another experiment, the fear of backlash was found that negatively predict agentic behavior success of women (self-promotion), which is mediated by reduced locomotion (locomotion refers to strive toward goals without inhibition, distractions, or delays) (Moss-Racusin and Rudman, 2010). Additionally, the fear of backlash is negatively related to the willingness of people to publicize their success under violation of racial stereotypes (Phelan and Rudman, 2010).

Based on the review of previous literature for women coping strategies for agency-communion tensions in the double-bind dilemma, we found that the research findings of experiments in virtual counter-stereotypical conditions and findings from the individual in real counter-stereotypical occupation were paradoxical. In experimental findings, women would hide success or increase gender conformity by diminish agentic behavior and raise communal behavior under stereotypical expectation violation conditions, which is motivated by fear of backlash (mediation effect) (Rudman and Fairchild, 2004; Moss-Racusin and Rudman, 2010; Rudman et al., 2012b). Nevertheless, women in real counter-stereotypical occupations would sometimes tend to increase gender conformity and sometimes tend to decrease gender conformity (Ely, 1995). We believe that this inconsistency is due to the individual difference that women perceive fear of backlash in various levels in a real occupation environment. For women in counter-stereotypical occupation, some may believe that they adapted to the male-dominant occupation pretty well, and they do not experience fear of backlash, whereas others may be more sensitive to the double-bind dilemma and experience a higher level of fear of backlash. The fear of backlash would function as more like a moderation factor in whether women increase or decrease gender conformity with real counter-stereotypical occupation.

In the current research, we selected the real occupation “senior business executives” as the counter-stereotypical role, and “lecturers” as a gender-neutral role, assuming that the high level of status and power of senior executives associated with the position and the low level of female representation therein would create a high level of conflict for women in terms of gender expectations and the potential double-bind dilemma, comparing to female lecturers.

Female senior executives who are in a male-dominant occupation that highly require competence and agency, may reinforce agency and reduce communion in the workplace. Therefore, female senior executives may describe themselves as more agentic and less communion than female lecturers who are in gender-neutral occupations. Previous researches provide evidence that the fear of backlash motivates counter-stereotypical women to increase gender conformity behavior by diminish agency and raise communion. The agency, as well as the communion difference between female senior executives and female lecturers, would be attenuated for female senior executives who highly fear potential backlash. Thus, we propound that fear of backlash may moderate whether female senior executives decrease or increase gender conformity (raise agency and diminish communion defined as decrease gender conformity; diminish agency and raise communion defined as increase gender conformity for women) compared with female lecturers.

We explore the following hypotheses and research questions including,

*Hypothesis 1: Female senior executives describe themselves as more agentic than female lecturers*.

*Hypothesis 2: Female senior executives describe themselves as less communal than female lecturers*.

*Research question 1: Is the positive relationship between women's role type (senior executives vs. lecturers) and agency attenuated by fear of backlash?*

*Research question 2: Is the negative relationship between women's role type (senior executives vs. lecturers) and communion attenuated by fear of backlash?*

### Overview of the Present Study

This study seeks to test whether female senior executives tend to increase gender conformity or decrease gender conformity (raise agency and diminish communion defined as decrease gender conformity; diminish agency and raise communion defined as increase gender conformity for women) as their coping strategy in response to the double-bind dilemma and explore the moderation effect of fear of backlash. Only female participants were selected because the double-bind dilemma usually arises in the careers of counter-stereotypical women.

**Figure 1:**
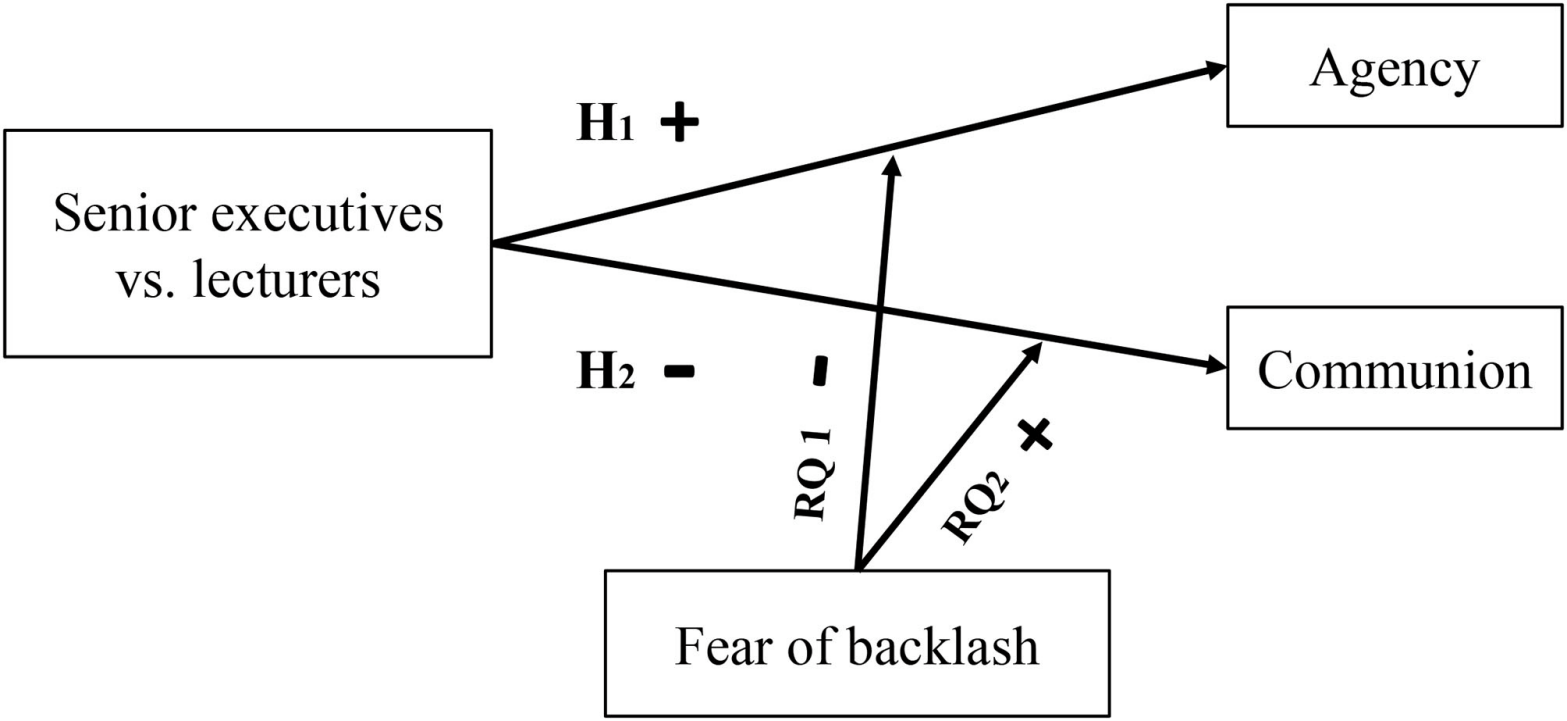
The path of hypotheses and research questions in the theoretical model. The “**+**” represents positive relationship and effect and “**–**” represents negative relationship and effect.

The current research primarily examined the prescriptive gender stereotype in China in Study 1 and provided agentic and communal attributes as research material for Study 2 and 3. Then, we tested whether female senior executives describe themselves as more agentic and less communal than female lecturers both under the imagination research paradigm (Study 2) and with women in actual occupations (Study 3) (Hypothesis 1 and 2). We further tested whether the positive relationship between the role type of women (senior executives vs. lecturers) and the agency was attenuated by fear of backlash and whether the negative relationship between the role type of women (senior executives vs. lecturers) and communion was attenuated by fear of backlash (Research question 1 and 2), as shown in Figure 1.

## Study 1

The purpose of Study 1 is to examine the current prescriptive gender stereotype in the Chinese context and provide agentic and communal attributes as research material in Studies 2 and 3. Before carrying out the research on whether there is a self-reported agency and communion difference between female senior executives and female lecturers and the moderation effect of fear of backlash, it is urgent to determine the belief of people about prescriptive gender stereotypes in Chinese society of today, and what attributes are desirable for men and women in the current Chinese context. Therefore, we display traits to participants to evaluate how stereotypical men or women are socially desired in China.

### Participants

A total of 212 university students (132 women, 80 men; *M*_age_ = 20.57, *SD* = 1.84) were involved in the exchange of course extra credit of an “Introductions to Psychology” course. They completed the study through the Chinese online survey platform Wenjuanxing. Concerning subject discipline, 19.8% of participants were from the science, technology, engineering, and mathematics (STEM) department, 73.1% were from humanities departments, and 7.1% were from medical science departments.

### Materials and Procedure

We adopted 49 attributes (including 23 words on masculinity and 26 words on femininity) from the new sex-role Inventory (Chinese sex role inventory, CSRI-50) and Bem Sex Role Inventory (Bem, 1974). New sex-role Inventory (Chinese sex role inventory, CSRI-50) was a widely used version of the Chinese sex-role inventory selected from 1,700 personality characteristics. Its reliability and validity were tested with 5,008 Chinese samples (Liu et al., 2011). In the current survey, we asked participants to “indicate how desirable it is in Chinese society for a woman [man] to have each of the following characteristics” on a scale from 1 (not at all desirable) to 9 (very desirable). Participants conducted the survey after reading the consent form and were debriefed after completing the survey.

### Prescriptive Gender Stereotypes

We defined male prescriptions as traits that were rated above six for men, and it had a greater gender difference effect size to *d* = 0.50 when compared with the desirability rating of men. As shown in Table 1, the male prescription traits were displayed on half top of the table (*M*_d_ = 0.82, range = 0.54–1.26). We define female prescriptions as traits that were rated above six for women, and it had less gender difference effect size to *d* = −0.50 compared with the desirability rating of women. As shown in Table 1, the female prescription traits were displayed on the half bottom of the table (*M*_d_ = −0.98, range = −1.13 to −0.68).

**Table 1:** Prescriptive traits for men and women in China in Study 1.

**Traits**	**Prescriptive**	**Male**	**Female**
	***d***	***M***	***M***
**Men's prescriptions**			
Forceful*	1.26	7.45	5.51
Willing to take risks*	0.93	7.00	5.47
Dominant*	0.92	7.22	5.66
Assertive*	0.89	6.90	5.30
Ambitious	0.87	7.39	6.00
Bold	0.87	7.20	5.79
Rational	0.68	7.00	5.95
Brave	0.64	7.24	6.28
Independent*	0.63	7.11	6.13
Generous	0.54	6.89	6.03
**Women's prescriptions**			
Soft-spoken	−1.13	5.28	7.29
Graceful and quiet	−1.1	5.04	6.92
Eager to soothe hurt feelings	−1.09	5.58	7.41
Tender*	−1.09	5.53	7.40
Shy	−1.06	5.07	6.88
Understanding	−1.05	5.63	7.34
Gentle	−1.01	5.56	7.21
Warm*	−0.97	5.21	6.94
Sympathetic	−0.96	5.74	7.30
Sensitive to others' need	−0.91	5.36	6.97
Compassionate*	−0.85	6.03	7.41
Affectionate*	−0.84	5.67	7.06
Loves children	−0.8	6.24	7.59
Good listener	−0.79	5.77	7.11
Yielding*	−0.75	5.26	6.58
Considerate	−0.68	5.91	7.05

*Participants were asked to “indicated how desirable it is in Chinese society for a woman [man] to have each of the following characteristics” on a scale from 1 (not at all desirable) to 9 (very desirable). The traits marked with asterisk were randomly selected as research material for Study 2 and Study 3. N = 212*.

### Results

In sum, the pilot study revealed the prescriptive stereotypes of men and women in Chinese society. As shown in Table 1, the prescriptive stereotypes of men displayed agency content, and prescriptive stereotypes of women displayed communion content. We randomly selected five attributes (independent, forceful, dominant, assertive, and willing to take risks, marked with an asterisk) representing agency (gender difference effect size greater than *d* = 0.50) and five attributes (yielding, affectionate, warm, tender, and compassionate, marked with an asterisk) representing communion (gender difference effect size less than *d* = −0.50) as experimental materials in the following Study 2 and 3.

## Study 2

Study 2 observed whether female senior executives describe themselves as more agentic (Hypothesis 1) and less communal (Hypothesis 2) than female lecturers at work using women university students as participants. We also explored whether the positive relationship between the role type of women (senior executives vs. lecturers) and the agency is attenuated by fear of backlash (Research question 1) and whether the negative relationship between role type (senior executives vs. lecturers) of women and communion is attenuated by fear of backlash (Research question 2). In Study 2, participants are imagining themselves in the role and assessing their self-reported agency, self-reported communion (traits selected from Study 1), and the fear of backlash at work under the imagination research paradigm.

### Method

#### Participants

A total of 207 female university students (*M*_age_ = 19.43, *SD* = 1.55) were recruited using participation recruitment posters in the university library. The students received 1 RMB as a reward for participating. They completed the experiment through the Chinese online survey platform Wenjuan Xing. Sensitivity analysis of power using G^*^Power indicated that the current sample size would produce a 95% chance of detecting a medium effect size of 0.25 in the one-way ANOVA between the two groups as significant at the 5% level (two-tailed) (Faul et al., 2007).

Concerning the subject discipline of participants, 18.4% of the participants were from the STEM departments, 62.3% were from humanities departments, and 19.3% were from medical science departments.

#### Procedure

Participants first read that the purpose of the experiment was to explore personal experience in different situations. They then indicated their informed consent by clicking the “I agree” checkbox. After being asked to confirm their gender, participants were randomly allocated to either the senior executive role condition or the lecturer role condition.

In the senior executive condition, participants received the following information and wrote down their answers before being asked to indicate the agentic and communal attributes: “Please imagine yourself as a senior executive, and list at least 10 daily activities that you would have to do as a senior business executive.” There was no gender difference in the Mandarin-language description for the position of a senior executive. So, the effect of the feminine or masculine form of language was not considered in the experiment (Horvath et al., 2016). In the lecturer condition, participants received the following information before indicating their agentic and communal attributes: “Please imagine yourself as a lecturer, and list at least 10 daily activities that you would have to do as a lecturer.” The answers from both groups of participants were checked to ensure that every participant had comprehended the instructions. After answered those questions, participants responded to items concerning their self-reported agency and communion at work (agentic and communal attributes from Study 1), fear of backlash, and finally provided demographic information.

#### Self-Reported Agency and Communion

The dependent variables were self-reported agentic and communal attributes at work. The participants in the two conditions were asked in sequences, “As a senior business executive/lecturer, to what extent are you to display such a trait at work?” Each attribute was rated on a 7-point scale, ranging from 1 (“not at all”) to 7 (“very much”). We measured the self-reported agency of women using five agentic words adopted from Study 1, encompassing “independent,” “forceful,” “dominant,” “assertive,” and “willing to take risks” (agentic attributes at work, α = 0.68). The self-reported communion was also measured using five communal words adopted from Study 1, covering “yielding,” “affectionate,” “warm,” “tender,” and “compassionate” (communal attributes at work, α = 0.85).

#### Fear of Backlash

The fear of backlash was measured by two items adapted from the work of Moss-Racusin and Rudman (2010), which was revised for the current study (The revision was based on interviewing three Chinese female senior executives). The items included “Would you worry that people might think you were not feminine enough?” and “Would you be concerned that you might be disliked?” The internal consistency reliability was acceptable (α = 0.79).

### Results

Table 2 presented the overall means, SDs, and correlations among the variables in Study 2.

**Table 2:** Descriptive statistics and correlations in Study 2.

**Variables**	***M*** **±*SD***	**1**	**2**	**3**	**4**	**5**	**6**
Age	19.43 ± 1.55	–					
Major	2.01 ± 0.62	−0.01	–				
Role type	0.50 ± 0.50	−0.03	0.02	–			
Agency at work	5.15 ± 0.93	0.16*	−0.10	0.22**	–		
Communion at work	3.95 ± 1.5	0.26**	−0.04	−0.60**	−0.01	–	
Fear of backlash	3.88 ± 1.36	−0.00	−0.02	0.04	0.11	0.15*	–

* *p < 0.05*,

** *p < 0.01. N = 207 women. Role type recoded as 1 = senior executive, 0 = lecturer*.

#### Self-Reported Agency and Communion and Fear of Backlash Difference Analysis in Study 2

**Figure 2:**
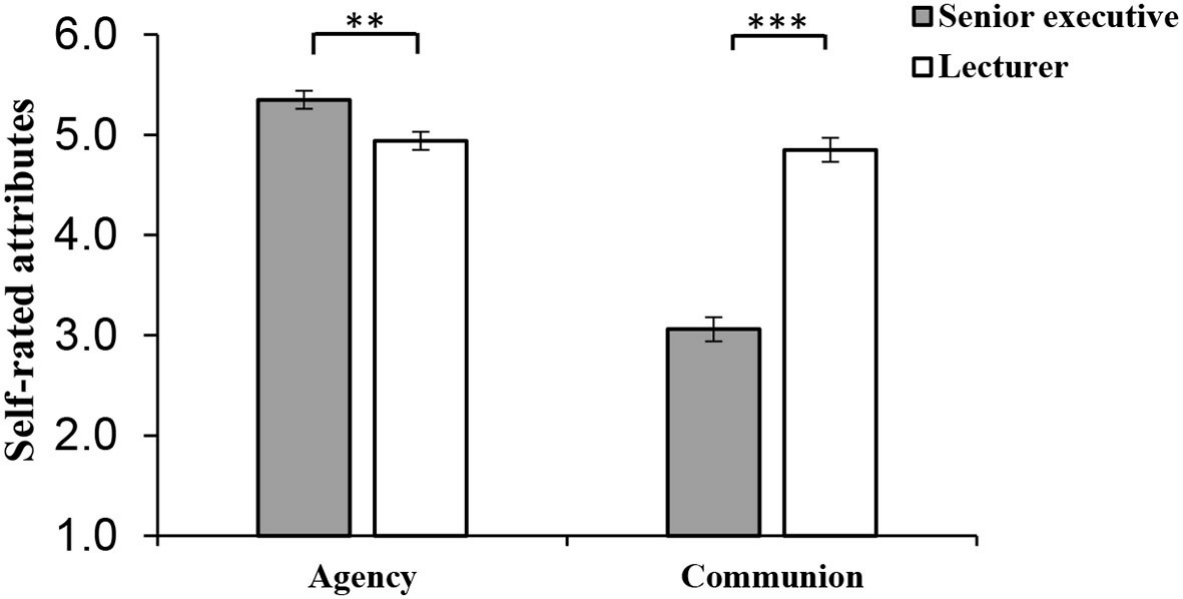
Self-reported agency and communion at work for female senior executives and female lecturers in Study 2 under imagination research paradigm. Error bars represent the SE of the mean. ***p < 0.001, **p < 0.01. *N* = 207 women (*N*_Executive_= 104, *N*_Lecturer_ = 103).

One-way ANOVA was undertaken to test Hypothesis 1 and 2 to determine whether female senior executives rated themselves more agentic and less communal than female lecturers. The results (shown in Figure 2) showed that female senior executives describe themselves as significantly more agentic (*M* = 5.35, *SD* = 0.84) than female lecturers (*M* = 4.94, *SD* = 0.98), *F*_(1, 205)_ = 10.16, *p* < 0.01, $\eta_{p}^{2}\;=$ 0.05 at work. Conversely, they reported significantly less communion at work (*M* = 3.06, *SD* = 1.38) than female lecturers (*M* = 4.85, *SD* = 1), *F*_(1, 205)_ = 113.79, *p* < 0.001, $\eta_{p}^{2}\;=$ 0.36. Meanwhile, fear of backlash of women in the senior executive condition had no difference with women in the lecturer condition (*p* = 0.61).

#### The Moderation Effect of Fear of Backlash on the Relationship Between Role Type and Self-Reported Agency and Communion in Study 2

The next section of the results was concerned with the moderation effect of fear of backlash on the relationship between role type and self-reported agency and communion (Research questions 1 and 2).

The hierarchical regression was employed to analyze whether fear of backlash moderated the relationship between occupation role type of women (role type recoded as 1 = senior executive, 0 = lecturer) and self-reported agency at work. The main effect is significant. The role type (β = 0.21, *p* < 0.01, 95% CI = [0.08, 0.35]) significantly positively predicted self-reported agency of women, which indicated that female senior executives describe themselves as more agentic than female lecturers at work. Further, the fear of backlash (β = 0.10, *p* = 0.16, 95% CI = [−0.04, 0.23]) was not significantly a predicted self-reported agency of women. The interaction effect of role types and fear of backlash was not significant either (β = 0.10, *p* = 0.16, 95% CI = [−0.04, 0.24]), which indicated that there was no moderation effect of fear of backlash on self-reported agency with the role types of women.

Then, we also used hierarchical regression to analyze the moderation effect of fear of backlash on the relationship between occupation role types of women (role type recoded as 1 = senior executive, 0 = lecturer) and their self-reported communion. It can be seen from Table 3 that in Model 1, role type (β = −0.60, *p* < 0.001, 95% CI = [−0.71, −0.50]) significantly negatively predicted the self-reported communion of women which demonstrated that female senior executives describe themselves as less communal than female lecturers, and fear of backlash (β = 0.17, *p* < 0.01, 95% CI = [0.06, 0.28]) significantly positively predicted the self-reported communion of women in Model 1. The interaction effect of role types and fear of backlash was significant (β = 0.15, *p* = 0.007, 95% CI = [0.04, 0.26]) in Model 2. The results illustrated that there is a positive interaction effect for the role type of women and fear of backlash on self-reported communion; the negative relationship (the difference between self-reported communion of senior executive and self-reported communion of lecturer) was weaker in individuals with high fear of backlash. Thus, the moderation effect of fear of backlash on self-rated communion of women (but not agency) with occupation role types was significant, and the results responded to Research questions 1 and 2.

**Table 3:** Hierarchical regression analyses for the moderator effect of fear of backlash on the relationship between role type of women and self-reported agency and communion in Study 2.

**Agency**	**Model 1**			**Model 2**		
	**β**	***SE***	***p***	**β**	***SE***	***p***
Role type	0.21	0.07	0.00	0.21	0.07	0.00
Fear of backlash	0.10	0.07	0.16	0.12	0.07	0.10
Role type × fear of backlash				0.10	0.07	0.16
*R^2^*		0.057**		0.066**		
Δ*F*		6.12		2.03		
**Communion**	**Model 1**			**Model 2**		
	***β***	***SE***	***p***	***β***	***SE***	***p***
Role type	−0.60	0.06	0.00	−0.60	0.05	0.00
Fear of backlash	0.17	0.06	0.00	0.20	0.06	0.00
Role type × fear of backlash				0.15	0.06	0.01
*R^2^*		0.385***		0.407***		
Δ*F*		63.81***		7.41**		

*p < 0.05,

** *p < 0.01*,

*** *p < 0.001. N = 207 women. Role type recoded as 1 = senior executive, 0 = lecturer, and all values of variables are Z-standardized*.

**Figure 3:**
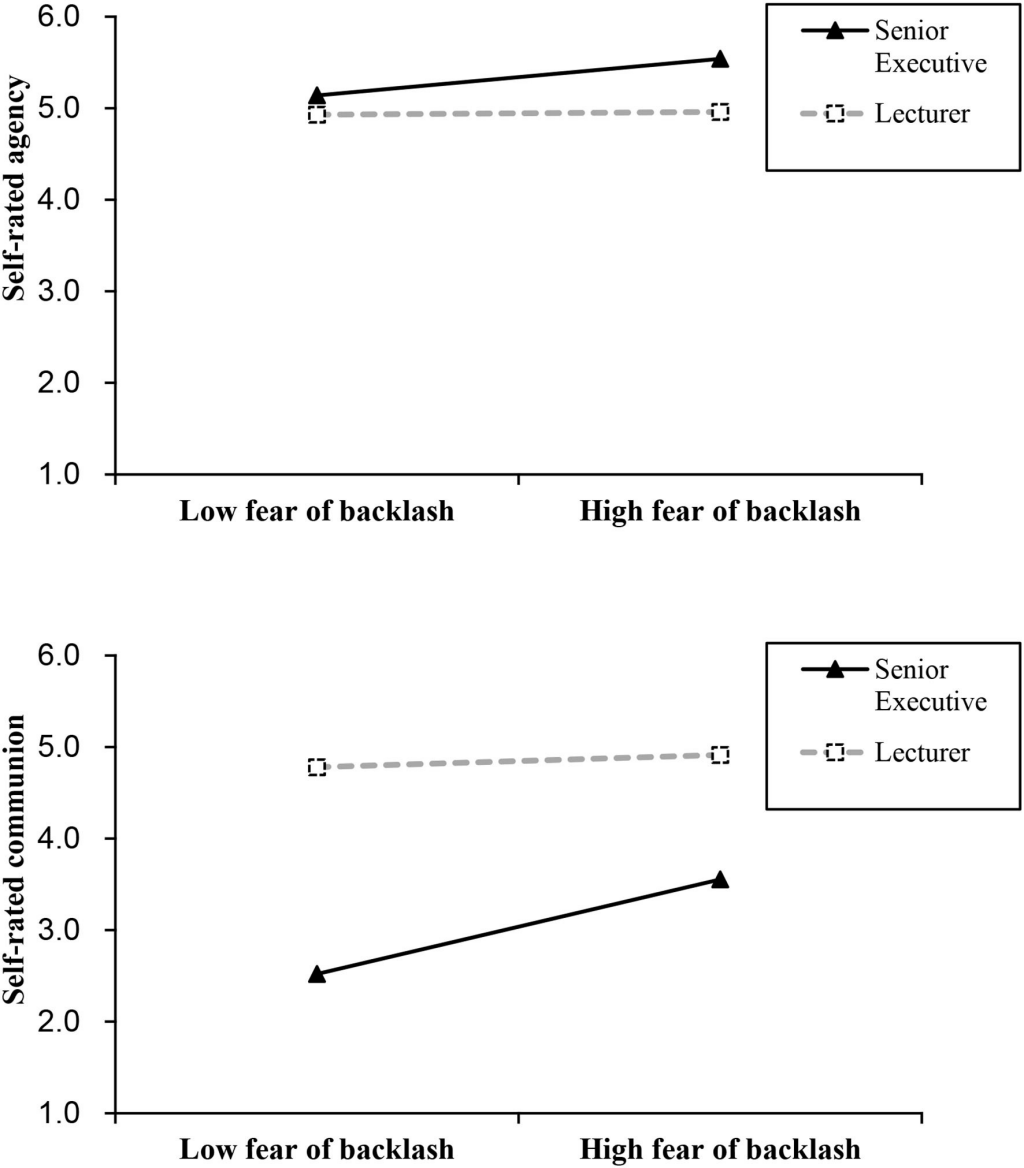
Simple slope analysis for moderation effect of fear of backlash on the relationship between the role type of women and self-reported agency and communion in Study 2 under imagination research paradigm. *N* = 207 women (*N*_Executive_= 104, *N*_Lecturer_ = 103).

We further performed simple slopes tests for exploring the difference of self-reported agency and communion between female senior executives and female lecturers with a lower fear of backlash (1 *SD* below the mean of fear of backlash) and women with a higher fear of backlash (1 *SD* above the mean of fear of backlash). The results are presented in Figure 3. The difference of self-reported agency between female senior executives and lecturers has a slight change from individuals with low fear of backlash (β = 0.12, *p* = 0.24, 95% CI = [−0.08, 0.31]) to individuals with high fear of backlash (β = 0.31, *p* < 0.01, 95% CI = [0.12, 0.50]). However, the difference of self-reported communion between female senior executives and female lecturers was significantly weaker from individuals with low fear of backlash (β = −0.76, *p* < 0.001, 95% CI = [−0.91, −0.60]) to individuals with high fear of backlash (β = −0.45, *p* < 0.001, 95% CI = [−0.61, −0.30]).

**Figure 4:**
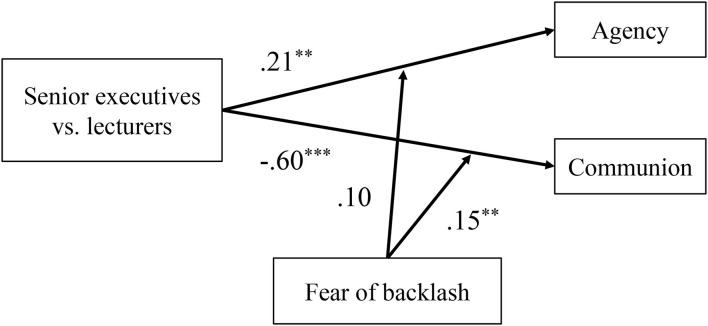
Standardized regression coefficient for moderation effect of fear of backlash between role types and self-reported agency and communion in Study 2. Role type was recoded as 1 = senior executive, 0 = lecturer. The “+” sign represents positive relationship and effect and —represents negative relationship and effect. All values of variables are *Z*-standardized. **p < 0.01, ***p < 0.001. *N* = 207 women.

Overall, as shown in Figure 4, the main effect of role type on the self-reported agency (β = 0.21^**^) and communion (β = −0.60^***^) was significant, which indicated that female senior executives described themselves as more agentic and less communal than female lecturers. The moderation effect of fear of backlash was only significant between role type and self-reported communion (but not agency) (β = 0.15^**^). It demonstrated that the negative relationship between role type of women and communion was attenuated by fear of backlash, and the self-reported communion difference between female senior executives and female lecturers was weaker in individuals with high fear of backlash.

### Discussion

The results of Study 2 support our prediction that female senior executives described themselves as more agentic (Hypothesis 1) and less communal (Hypothesis 2) at work than female lecturers. Besides, we also found that fear of backlash attenuated the effect of role type on self-reported communion of women (but not agency) (responded to Research questions 1 and 2).

It could be argued that the results of Study 2 are due to the imagination research paradigm. The imagination of female university students may exaggerate the self-reported agency and communion difference between female senior executives and female lecturers according to their stereotypical beliefs. Therefore, we further examine our hypotheses and research questions in Study 3 with participants in real senior executive and lecturer roles.

## Study 3

### Method

#### Participants

The study sample of 202 participants was made up of 96 female senior executives and 106 female lecturers employed in Hubei Province, China. Using the Hubei Enterprise Management Talents Association's email list, we randomly selected 200 female senior executives who were managers, directors, or vice presidents in businesses and local government-affiliated institutions. The response rate was 48%, and 96 female senior executives completed the questionnaire. Two hundred female lecturers were randomly selected from the lecture email list of universities in Hubei Province, and the response rate was 53%, and 106 female lecturers completed the questionnaire. Sensitivity analysis of power using G^*^Power indicated that the current sample size would produce a 94% chance of detecting a medium effect size of 0.25 in the one-way ANOVA between the two groups as significant at the 5% level (two-tailed) (Faul et al., 2007).

The age of participants ranged from 24 to 70 years, with a mean age of 41.81 (*SD* = 8.93), and the median age was 41 years. The mean working period of participants as senior executives or lecturers was 14.75 years (*SD* = 8.63). The education levels of the participants varied, with 1% of them having a high school graduation degree, 43.1% had a bachelor's degree, 37.1% had a master's degree, and 18.8% held a doctoral degree. Regarding their marital status, 12.9% were unmarried, 78.7% were married, and 8.4% had divorced.

#### Procedure and Measures

The participants accessed the online study *via* a link on the recruitment email. They were informed that the purpose of the study was to explore personal working experiences. They then indicated their informed consent by clicking the “I agree” checkbox. As similar in Study 2, participants completed the following measures: gender confirmation question, self-reported agency and communion at work, and fear of backlash. In the end, they provided demographic information.

#### Self-Reported Agency, Communion, and Fear of Backlash

The measured variables, including agentic and communal attributes, were the same as Study 2. The participants were asked in sequence, “As a senior business executive/lecturer, to what extent are you to display such a trait at work?” Each attribute was rated on a 7-point scale, ranging from 1 (“not at all”) to 7 (“very much”). The internal consistency of those measures was acceptable (agentic attributes at work, α = 0.78; communal attributes at work, α = 0.73; fear of backlash, α = 0.80).

### Results

Table 4 presented overall means, SDs, and correlations for women in different types of roles in Study 3.

**Table 4:** Descriptive statistics and correlations in Study 3.

**Variables**	***M*** **±*****SD***	**1**	**2**	**3**	**4**	**5**	**6**	**7**	**8**
Age	41.81 ± 8.93	–							
Education levels	2.74 ± 0.77	−0.04	–						
Working period	14.75 ± 8.63	0.75**	0.03	–					
Marital status	1.96 ± 0.46	0.33**	−0.08	0.28**	–				
Role type	0.48 ± 0.50	0.25**	−0.26**	−0.05	0.16*	–			
Agency at work	5.07 ± 0.98	0.10	−0.03	0.04	0.19**	0.30**	–		
Communion at work	4.68 ± 0.92	−0.10	0.13	0.04	−0.02	−0.31**	0.04	–	
Fear of backlash	3.68 ± 1.54	0.06	−0.18*	−0.00	0.02	0.14*	0.06	0.17*	–

* *p < 0.05*,

** *p < 0.01. N = 202 women. Role type recoded as 1 = senior executive, 0 = lecturer*.

#### Self-Reported Agency and Communion and Fear of Backlash Difference Analyses in Study 3

**Figure 5:**
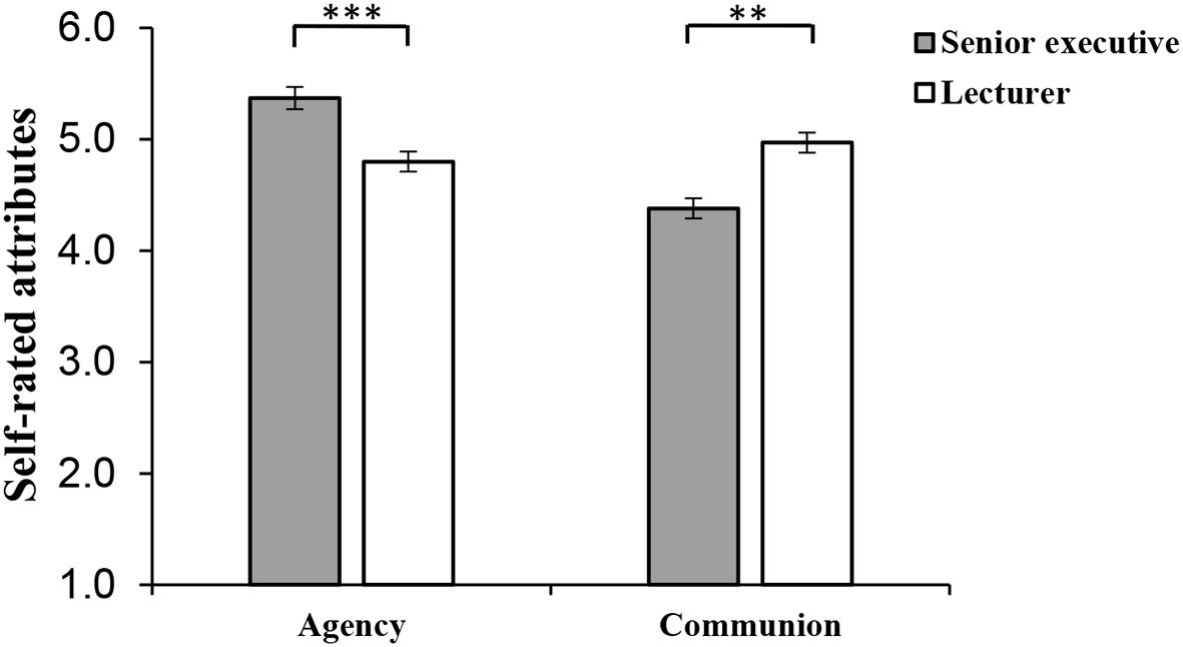
Self-reported agency and communion at work for female senior executives and women lecturers in Study 3. Error bars represent the standard error of the mean. ***p < 0.001, **p < 0.01. *N* = 202 women (*N*_Executive_= 96, *N*_Lecturer_ = 106).

We conduct an ANOVA analysis for self-reported agency and communion between female senior executives and female lecturers testing Hypothesis 1 and 2 (controlling demographic variables as covariates) (see Figure 5). The results showed that female senior executives described themselves more agentic (*M* = 5.37, *SD* = 0.93) than female lecturers (*M* = 4.79, *SD* = 0.95) at work, *F*_(1, 196)_ = 16.5, *p* < 0.001, $\eta_{p}^{2}\;=$ 0.08; conversely, female senior executives described themselves less communal (*M* = 4.38, *SD* = 0.92) than female lecturers (*M* = 4.95, *SD* = 0.83) at work, *F*_(1, 196)_ = 11.91, *p* < 0.01, $\eta_{p}^{2}\;=$ 0.06. Meanwhile, fear of backlash of female senior executives was in no way different from women lecturers (*p* = 0.30).

#### The Moderation Effect of Fear of Backlash on the Relationship Between Role Type and Self-Reported Agency and Communion in Study 3

The hierarchical regression was utilized to analyze whether fear of backlash moderated the relationship between women occupation role types (role type recoding similar as Study 2) and self-reported agency at work (controlling demographic variables as covariates). The main effect is significant, where the role types (β = 0.30, *p* < 0.001, 95% CI = [0.16, 0.43]) significantly positively predicted self-reported agency of women, which indicated that female senior executives reported more agency than female lecturers. Moreover, the fear of backlash (β = 0.01, *p* = 0.85, 95% CI = [−0.12, 0.15]) was not significantly predicted the self-reported agency of women. The interaction effect of role types and fear of backlash was not significant either (β = 0.02, *p* = 0.70, 95% CI = [−0.11, 0.16]), which indicated that there is no moderation effect of fear of backlash on self-reported agency with the role types of women.

Then, we used hierarchical regression to analyze the moderation effect of fear of backlash on the relationship between role type of women (role type recoding similar as Study 2) and their self-reported communion (controlling demographic variables as covariates). As shown in Table 5, role types (β = −0.34, *p* < 0.001, 95% CI = [−0.47, −0.21]) significantly negatively predicted the self-reported communion of women, whereas the fear of backlash (β = 0.22, *p* < 0.01, 95% CI = [0.09, 0.35]) significantly positively predicted the self-reported communion of women in Model 1. The significant main effect demonstrated that female senior executives describe themselves as less communal than female lecturers.

**Table 5:** Hierarchical regression analyses for the moderation effect of fear of backlash on the relationship between role type of women and self-reported agency and communion in Study 3 (controlling demographic variables as covariates).

**Agency**	**Model 1**			**Model 2**			**Model 3**		
	**β**	***SE***	***p***	**β**	***SE***	***p***	**β**	***SE***	***p***
Role type	0.30	0.07	0.00	0.30	0.07	0.00	0.32	0.08	0.00
Fear of backlash	0.01	0.07	0.85	0.02	0.07	0.83	0.02	0.07	0.84
Role type × fear of backlash				0.03	0.07	0.69	0.02	0.07	0.73
Age							−0.08	0.12	0.51
Education levels							0.05	0.07	0.45
Working period							0.08	0.11	0.47
Marital status							−0.11	0.07	0.12
*R^2^*		0.098***			0.090***			0.115**	
Δ*F*		9.78			0.16			1.37	
**Communion**	**Model 1**			**Model 2**			**Model 3**		
	***β***	***SE***	***p***	***β***	***SE***	***p***	***β***	***SE***	***p***
Role type	−0.34	0.07	0.00	−0.34	0.07	0.00	−0.29	0.08	0.00
Fear of backlash	0.22	0.07	0.00	0.23	0.07	0.00	0.24	0.07	0.00
Role type × fear of backlash				0.15	0.07	0.03	0.15	0.07	0.03
Age							−0.17	0.11	0.12
Education levels							0.07	0.07	0.31
Working period							0.14	0.11	0.21
Marital status							−0.08	0.07	0.28
*R^2^*		0.145***			0.165***			0.181***	
Δ*F*		16.82***			4.90*			0.91	

* *p < 0.05*,

** *p < 0.01*,

*** *p < 0.001. N = 202 women. Role type recoded as 1 = senior executive, 0 = lecturer, marital status recoded as 1 = married, 0 = not married (divorced or never married), and all values of variables are Z-standardized*.

In the Model 2, the interaction effect of role types and fear of backlash was significant (β = 0.15, *p* < 0.05, 95% CI = [0.02, 0.28]). In Model 3, the interaction effect of role types and fear of backlash were also significant under demographic controlled variables (β = 0.15, *p* < 0.05, 95% CI = [0.02, 0.28]). The results revealed that there is a positive interaction effect of role type and fear of backlash of women on self-reported communion, and the negative relationship between role type and self-reported communion is attenuated by fear of backlash. Thus, the moderation effect of fear of backlash on self-reported communion (but not agency) of women with different role types was significant, and the results responded to Research questions 1 and 2.

**Figure 6:**
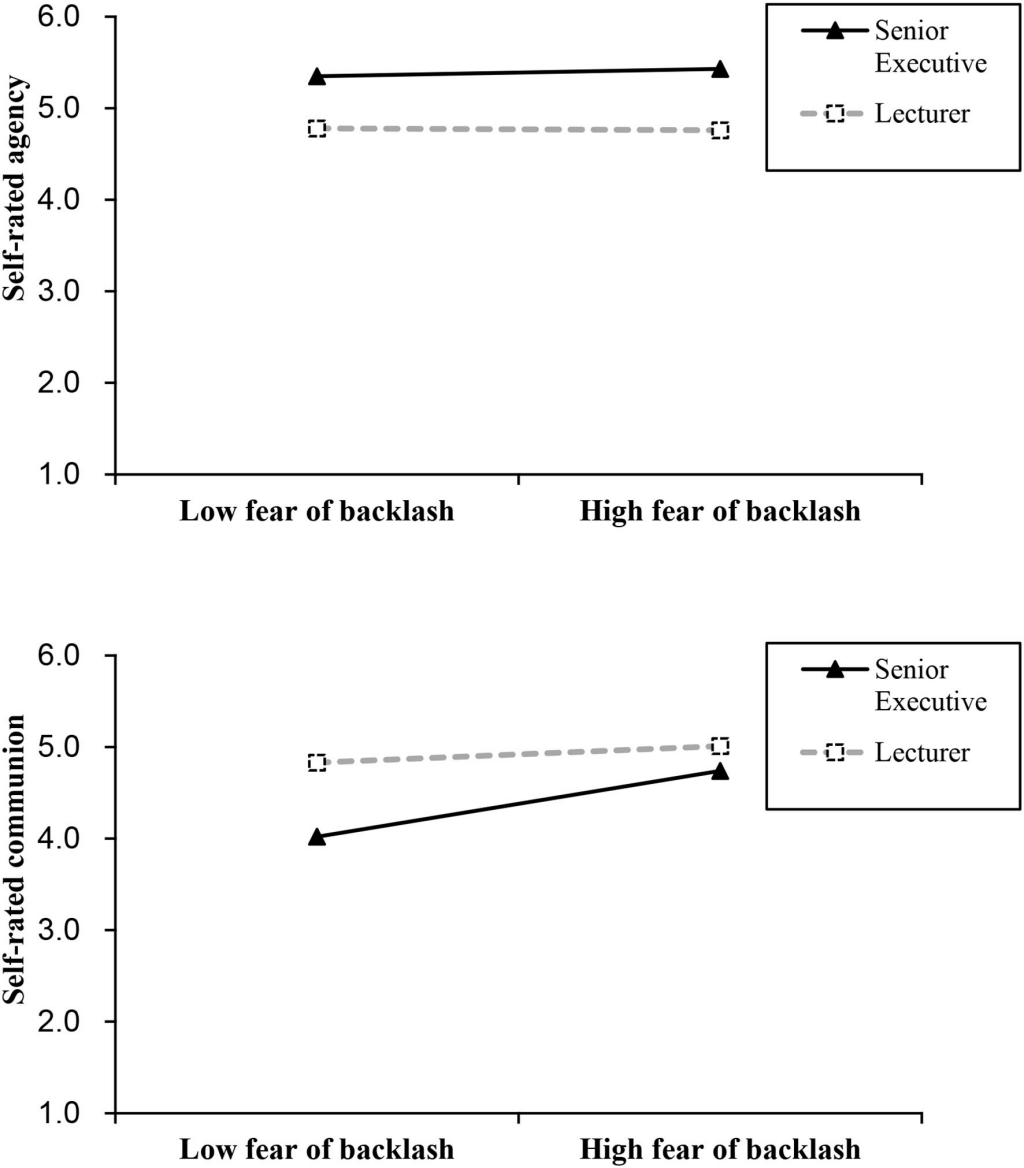
Simple slope analysis for moderation effect of fear of backlash on the relationship between the role type of women and self-reported agency and communion in Study 3. *N* = 202 women (*N*_Executive_= 96, *N*_Lecturer_ = 106).

Further simple slopes tests were probed for comparing the difference of self-reported communion between role types for women who had a lower fear of backlash (1 *SD* below the mean of fear of backlash) and women who had a higher fear of backlash (1 *SD* above the mean of fear of backlash). The results (in Figure 6) illustrated that the difference of self-reported agency between female senior executives and lecturers did not significantly change from individuals with low fear of backlash (β = 0.29 *p* < 0.01, 95% CI = [0.08, 0.50]) to individuals with high fear of backlash (β = 0.34, *p* < 0.01, 95% CI = [0.13, 0.55]). However, the difference of self-reported communion between female senior executives and female lecturers was weaker from individuals with low fear of backlash (β = −0.44, *p* < 0.001, 95% CI = [−0.64, −0.24]) to individuals with high fear of backlash (β = −0.14, *p* = 0.15, 95% CI = [−0.34, 0.06]).

**Figure 7:**
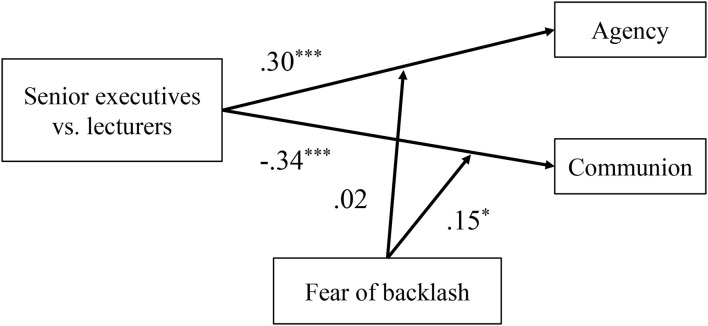
Standardized regression coefficient for moderation effect of fear of backlash between role types and self-reported agency and communion in Study 3. Role type recoded as 1 = senior executive, 0 = lecturer. The “+” represents positive relationship and effect and —represents negative relationship and effect. *p < 0.05, ***p < 0.001. *N* = 202 women.

Overall, the main effect of role type on the self-reported agency (β = 0.30^**^) and communion (β = −0.34^**^) was significant, which indicated that female senior executives described themselves as more agentic and less communal than female lecturers in Study 3 (see Figure 7). The moderation effect of fear of backlash was only significant between role type and self-reported communion (β = 0.15^*^) (but not agency), which demonstrated that the negative relationship between role type of women and self-reported communion was attenuated by fear of backlash.

### Discussion

In sum, Study 3 yields two main findings. First of all, we examined the self-reported agency and the communion of real female senior executives and female lecturers at work. The results support Hypothesis 1 and 2. Female senior executives generally reported more agency and less communion at work than female lecturers.

Second, the finding of Research questions 1 and 2 were consistent with Study 2. We found that the fear of backlash attenuated the effect of role type on the self-reported communion (but not agency) of women. However, the moderation effect pattern is different from Study 2. Under a lower level of fear of backlash, role types significantly predicted self-reported communion since the self-reported communion of female senior executives was much lower than that of female lecturers. In contrast, under a higher level of fear of backlash, the role type of women could not predict self-reported communion because there was no difference between self-reported communion of women senior executives and self-reported communion of women lecturers.

## General Discussion

This study set out to explore whether women in counter-stereotypical roles, such as senior executives, would decrease or increase gender conformity by self-reported agency and communion for coping agency-communion tensions in the double-bind dilemma and discover the moderation effect of fear of backlash. We primarily examined the prescriptive gender stereotype in China and identified the agentic and communal attributes that are desirable in the Chinese context for men and women. Then, we predict that female senior executives would generally have less gender conformity by self-reported more agency and less communion at work than female lecturers (Hypothesis 1 and 2). We also tried to discover whether the fear of backlash attenuated higher agency of female senior executives over lecturers and lower communion of female senior executives beyond lecturers at work.

The results of the three studies, which featured the use of an imagination research paradigm and also participants in the real counter-stereotypical role, support our hypothesis that female senior executives self-reported more agency and less communion at work than female lecturers in the Chinese context (Hypothesis 1 and 2). Also, there is no difference between the score on fear of backlash for women in the counter-stereotypical role and women in the gender-neutral role. Next, we tested how exactly the fear of backlash moderates strategies of female senior executives coping agency and communion tensions.

The results demonstrated that both in the imagination research paradigm and in real occupation, the fear of backlash attenuated the effect of role type on self-reported communion (but not agency) (Research questions 1 and 2). To be specific, the self-reported communion difference between female senior executives and female lecturers was shrunk when women have a higher level of fear of backlash. In real occupation roles, senior executives show no difference in self-reported communion with female lecturers even under a higher level of fear of backlash.

### Theoretical Implications

The present study answered the question about how counter-stereotypical women would react to the career barriers like the double-bind dilemma. In our research, female senior executives generally reported higher levels of agency and lower levels of communion than female lecturers. However, they reconcile to the agency–communion tension by having higher levels of agency and communion (almost as much communion as lecturers did) under the motivation of high fear of backlash. Accordingly, our study contributes to the literature of research on counter-stereotypical women in the double-bind dilemma for the following four aspects.

Firstly, current research explored the extent of prescriptive gender stereotypes in China and made the first attempt to verify coping strategies of female senior executives for the double-bind dilemma using agentic and communal attributes (prescriptive gender stereotype) as dependent variables in the Chinese context.

Second, our research initially confirms that Chinese female senior executives differ from female lecturers in self-reported agency and communion. Although there is some evidence which illustrates that women in the male-dominant environment would devalue femininity by exaggerating gender differences, avoid displaying feminine characteristics, and rate themselves less favorably (Ely, 1995; Larsson and Aida, 2020), the current study provides first-hand quantitative results on how women self-reported more agency and less communion in male-dominant occupation in the Chinese context.

Third, current results supplied empirical evidence that fear of backlash functions as a moderation factor that influences how female senior executives dealing with agency-communion tensions in both imagination scenarios and real occupation roles. This finding provides a bridge to understand the paradox results between virtual counter-stereotypical condition experiments and study focus on real experience in the counter-stereotypical occupation. The results demonstrated that there is an individual difference in how women perceive fear of backlash in real-world male-dominant occupations. Not every woman senior executive is sensitive to the double-bind dilemma and feels fear of backlash. In research of interviews with 76 mid-upper-level female managers across different countries, only one-third of managers mention that they are aware of the double-bind dilemma in the workplace (Peus et al., 2015). Furthermore, these findings contribute to the literature of counter-stereotypical women's subjective experience and explain why previous literature shows that some women may decrease gender conformity, whereas others reported that women would increase gender conformity in the double-bind dilemma (Roberts, 2005; Rudman et al., 2012b). The moderation effect of fear of backlash illustrated that women who are particularly sensitive to the double-bind and who are in a role where the double bind is relevant (e.g., senior executives) seek to make a compromise with regard to both roles (woman vs. executive) by increasing their communion and preserve the level of agency.

Lastly, we found that fear of backlash only moderates the communion of female senior executives, but it did not moderate the agency of female senior executives. These results deepen the comprehension of counter-stereotypical women's subjective sights of agency-communion tensions in the double-bind dilemma. Although agency and communion traits naturally seem as opposite attributes, women who are in a role where the double-bind is relevant will not necessarily treat agentic and communal behavior as paradoxical behavior. Under high fear of backlash, the efforts that female senior executives increase gender conformity to avoid negative impression does not have to diminish agency and raise communion at the same time. It implies that women in the double-bind dilemma have a flexible coping strategy for agency-communion tensions. The research of interview top women leaders also supports this idea that they invent different strategies to manage the tensions between agency and communion by viewing agency and communion as non-contradictions (Zheng et al., 2018b).

### Limitations and Future Directions

Our research has limitations and also implied directions for future studies.

Primarily, although we found that women in the counter-stereotypical role may increase communion under a higher fear of backlash, we did not investigate whether this strategy benefits women. On the one hand, it is unknown whether the coping strategy of women enhances their well-being. The researcher has already shown that counter-stereotypical women, such as female leaders, scientists, law students, and police officers may experience identity conflict, lower subjective well-being, and lower performance effectiveness when lacking an efficient coping strategy for the double-bind dilemma (McIntosh et al., 1994; Settles, 2004; Karelaia and Guillén, 2014; Veldman et al., 2017). On the other hand, it is uncovered whether this strategy improves the counter-stereotypical job performance and career success of women. Recent research demonstrated that this strategy would increase the leadership effectiveness of female senior executives because communal motive benefits the leadership ratings of women leaders and decreases the unethical decisions in business (Kennedy and Kray, 2014; Wolff and Keith, 2019). However, high leadership effectiveness does not ensure career success for women. There is less than 10% of managers had both successful career and leadership effectiveness, and communal motives were unrelated with leadership role occupancy (Kaiser et al., 2008; Wolff, 2019, p. 68–69). Therefore, future research might also benefit from having a differentiated view on communion as being yielding can both be very effective or ineffective depending on various aspects of the career path of women.

Second, the current research only examines counter-stereotypical behavior patterns of women in the context of Chinese culture. Previous qualitative research has indicated that women leaders in the United States more frequently mentioned the double-bind dilemma than Chinese women leaders (Peus et al., 2015). Other evidence suggests that individuals in China embrace lower implicit prescriptive gender stereotypes about women vs. men, and the current promotion of gender equality in China facilitated a more liberal orientation toward the gender role of women (Zuo and Liu, 2006; Shu and Zhu, 2012, p. 1103). It is still unknown whether Chinese women leaders experience a lower level of the double-bind dilemma than American women leaders. Crosscultural studies of counter-stereotypical women and their behavior patterns are therefore encouraged.

Last, we only examined the coping strategy for women in the role of senior executives. As we stated previously in the discussion session, women may experience different fear of backlash in the different counter-stereotypical roles. Future studies are needed to test whether women in other counter-stereotypical roles are increasing or decreasing conformity under fear of backlash and what factors contribute to people perceived a higher level of fear of backlash.

All in all, fear of backlash would indeed change the coping strategy of women for the agency-communion tension, especially for individuals who are sensitive to the double-bind situation. Counterintuitively, a lower level of the communion of female senior executives could be the consequence of self-selection, and a high level of communion could be a coping strategy under the pressure of gender stereotypes. It implied that it is vital to change gender expectations in male-dominant occupations, especially gender expectations which contradict with occupation stereotype, for women to fulfill career development.

## Data Availability Statement

The datasets generated for this study are available on request to the corresponding author.

## Ethics Statement

The studies involving human participants were reviewed and approved by the Ethics Committee of Center for Studies of Social Psychology at Central China Normal University. The patients/participants provided their written informed consent to participate in this study.

## Author Contributions

XT, BZ, and FW conceived and designed the study. BZ and XT collected the data. XT analyzed, interpreted the data, and wrote the manuscript. XT, BZ, FW, ZX, and SS contributed to the revision process of the manuscript. All authors contributed to the article and approved the submitted version.

## Funding

This study was funded by the Major Program of National Social Science Foundation of China (18ZDA331). This research was also supported by grants from Young Scholar Grant 31800941 from the National Natural Science Foundation of China.

## Conflict of Interest

The authors declare that the research was conducted in the absence of any commercial or financial relationships that could be construed as a potential conflict of interest.

## Publisher's Note

All claims expressed in this article are solely those of the authors and do not necessarily represent those of their affiliated organizations, or those of the publisher, the editors and the reviewers. Any product that may be evaluated in this article, or claim that may be made by its manufacturer, is not guaranteed or endorsed by the publisher.
